# A Muti-Substrate Flavonol *O*-glucosyltransferases from Safflower

**DOI:** 10.3390/molecules28227613

**Published:** 2023-11-15

**Authors:** Shuyi Qi, Beixuan He, Haotian Wang, Yaqian Duan, Lunuan Wang, Yue Gao, Meili Guo

**Affiliations:** 1 Department of Pharmacognosy, College of Pharmacy, Naval Medical University (Second Military Medical University), Shanghai 200433, China; 2 Chemistry Experimental Teaching Center, College of Pharmacy, Naval Medical University (Second Military Medical University), Shanghai 200433, China; dyq37579@163.com; 3 Changhai Clinical Research Unit, The First Affiliated Hospital of Naval Medical University (Second Military Medical University), Shanghai 200433, China

**Keywords:** safflower, *O*-glycosyltransferase, flavonoid, biosynthesis

## Abstract

To explore the complete biosynthesis process of flavonoid glycosides in safflower, specifically the key glycosyltransferase that might be involved, as well as to develop an efficient biocatalyst to synthesize flavonoid glycosides, a glycosyltransferase *Ct*UGT4, with flavonoid-*O*-glycosyltransferase activity, was identified in safflower. The fusion protein of *Ct*UGT4 was heterologously expressed in *Escherichia coli*, and the target protein was purified. The recombinant protein can catalyze quercetin to form quercetin-7-*O*-glucoside, and kaempferol to form kaempferol-3-*O* in vitro, and a series of flavones, flavonols, dihydroflavones, chalcones, and chalcone glycosides were used as substrates to generate new products. *Ct*UGT4 was expressed in the tobacco transient expression system, and the enzyme activity results showed that it could catalyze kaempferol to kaempferol-3-*O*-glucoside, and quercetin to quercetin-3-*O*-glucoside. After overexpressing *CtUGT4* in safflower, the content of quercetin-3-*O*-rutinoside in the safflower florets increased significantly, and the content of quercetin-3-*O*-glucoside also tended to increase, which preliminarily confirmed the function of *Ct*UGT4 flavonoid-*O*-glycosyltransferase. This work demonstrated the flavonoid-*O*-glycosyltransferase function of safflower *Ct*UGT4 and showed differences in the affinity for different flavonoid substrates and the regioselectivity of catalytic sites in safflower, both in vivo and in vitro, providing clues for further research regarding the function of *UGT* genes, as well as new ideas for the cultivation engineering of the directional improvement of effective metabolites in safflower.

## 1. Introduction

Flavonoids are important bioactive components in medicinal plants and exhibit a variety of pharmacological behaviors, including antioxidant activity [1], antiviral activity [2], antidepressant-like activity [3], antibacterial activity [4], preventative activity against the risk of coronary heart disease [5], anti-inflammatory activity, and anticancer activity [1,2,3,4,5,6,7]. In response to these therapeutic properties, flavonoids derived from various medicinal plants offer a wide range of therapeutic applications for humans [8]. Numerous studies have gradually revealed the biosynthesis of these flavonoids in their respective medicinal plants of focus. Increasingly, researchers have also focused on various strategies for enhancing the content of active flavonoids in medicinal plants. The rapid development of biotechnology offers the promise of industrial biosynthesis using specific flavonoid-rich plants or yeast cells [9].

Glycosyltransferases, enzymes necessary for glycosylation reactions, are widely available in plants, often catalyzing the last step in the synthesis of plant secondary metabolites. A large number of glycosyltransferases have been reported so far. The carbohydrate-active enzyme database CAZy (CAZy, http://www.cazy.org, accessed on 1 April 2023) divides known glycosyltransferases into 110 families, among which the GT1 superfamily boasts the most members, and enzymes involved in the glycosyl transfer of plant secondary metabolism are assigned to this superfamily [10]. The specific role of the GT1 family is to transfer nucleotide-diphosphate (UDP) -sugars to low-molecular-weight substrates [11], so they are also called UDP-glycosyltransferases (*UGT*s). *UGT* family genes possess similar protein structures, and they all exhibit a highly conserved plant secondary product glycosyltransferase (PSPG)-box amino acid region for binding UDP moiety of UDP-sugars. According to the difference between the transferred sugar moiety and the substrate, there are 71 to 100 plant *UGT* subfamilies [12,13,14]. Enzyme-mediated glycosylation reactions are characterized by substrate specificity and high efficiency, which can alter the hydrophilicity, stability, chemical properties, subcellular localization, and biological activity of the receptors [15]. At present, some *UGT*s with flavonoids as substrates have been reported in medicinal plants, including the flavone 7-*O*-glucosyltransferase *Sb*UBGT in *Scutellaria baicalensis* and the 3-*O*-glycosylation *Sb*3GT1 of flavonols and anthocyanins [16,17,18], Pueraria’s isoflavone 7-*O*-glucosyltransferases *Pl*UGT1, *Pl*UGT4, *Pl*UGT15, and *Pl*UGT57, etc. [19,20,21]. In addition to participating in plant secondary metabolism as an important component, UGTs have shown great potential as bio-catalysts in the field of synthetic biology. Arend et al. recombinantly expressed the glycosyltransferase extracted from plant cell suspensions in bacteria and increased the expression level by 1800-fold compared to that of the plant system [22]. Lim et al. found that UGT, as a regioselective biocatalyst, can recognize a wider range of compounds in vitro than that noted in plants [23]. From the mining of *UGT* genes to the verification of UGT enzymatic functions, the important role of the *UGT* family has been widely recognized.

Safflower (*Carthamus tinctorius* L.) is an important traditional Chinese medicinal plant that activates blood circulation and removes stasis, and it is used in modern medicine to prevent and treat cardiovascular and cerebrovascular diseases [24]. Safflower’s pharmacological activities chiefly depend on its secondary metabolites, flavonoids. The flavonoids in safflower mainly accumulate in the flowers and are divided into quinone chalcones and flavonols. The representative components include Hydroxysafflor Yellow A, carthamin, kaempferol-3-*O*-rutinoside, quercetin-3-*O*-glucoside, etc. [25]. Most of these flavonoids are flavonoid glycoside forms, which exhibit pharmacological activities such as dilating blood vessels, improving microcirculation, preventing inflammation, and counteracting tumors and viruses [26,27,28,29,30]. Therefore, the exploration of flavonoid glycosides in safflower, especially to determine their biosynthetic pathways, is of great significance for selecting and breeding high-quality safflower varieties and efficiently targeted the production or industrial preparation of target-active ingredients.

The biosynthesis of the main active ingredients quinone chalcone glycosides and flavonol glycosides in the medicinal safflower plant is regulated by *UGTs*, but only a few *UGT* genes have been cloned in safflower [31]. *Ct*UGT84A33, *Ct*UGT71AE1, and *Ct*UGT90A14 were reported with isoquinoline alkaloid glycosylation activities [32]. The *CtUGT88B2* gene has been confirmed, through in vitro experiments [33], to exhibit an *O*-GT function. *Ct*UGT73AE1 shows the hybrid catalytic activity of *O*-GT, *N*-GT, and *S*-GT [34]. *Ct*UGT71E5 exhibits *N*-GT effects on various aromatic amine compounds [35]. However, most of the UGTs in safflower have not been clearly elucidated. Specifically, the UGTs that can directly regulate the flavonoid content in safflower is largely unknown.

In order to investigate the UGTs responsible for biosynthesizing flavonoid glycosides in safflower, the UGTs involved in the biosynthesis of flavonoid compounds in safflower were screened out. Subsequently, in vivo and in vitro functional validation experiments using identified UGTs were performed. The following is our first report of this study.

## 2. Results

### 2.1. Screening Candidate UGTs Regulating Flavonoid Biosynthesis Using Differential Circadian Rhythm Genes in Full Flower Stage

Since the content of the active ingredients in safflower varies within a day of its harvesting time, our research team preliminarily detected the flavonoid content of safflower at different times of the day and found that most of the flavonoids in safflower were significantly different between 9:00 and 15:00, so we analyzed the differential genes in the transcriptome database at these two timepoints.

**Figure 1:**
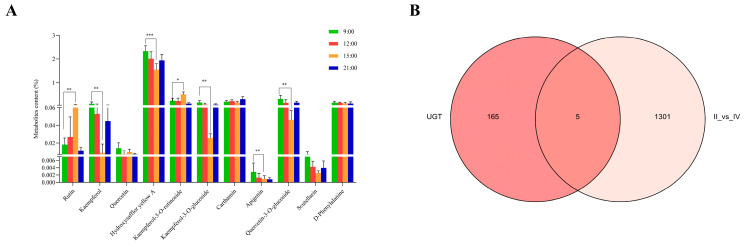
Screening of *UGT*s genes based on differences in accumulation of flavonoids in safflower at different timepoints of the day; (**A**) accumulation of flavonoid metabolites in safflower at four timepoints in one day (* *p* ≤ 0.05; ** *p* ≤ 0.01, *** *p* ≤ 0.001); (**B**) *UGT* gene showing significant expression difference between 9:00 and 15:00.

**Figure 2:**
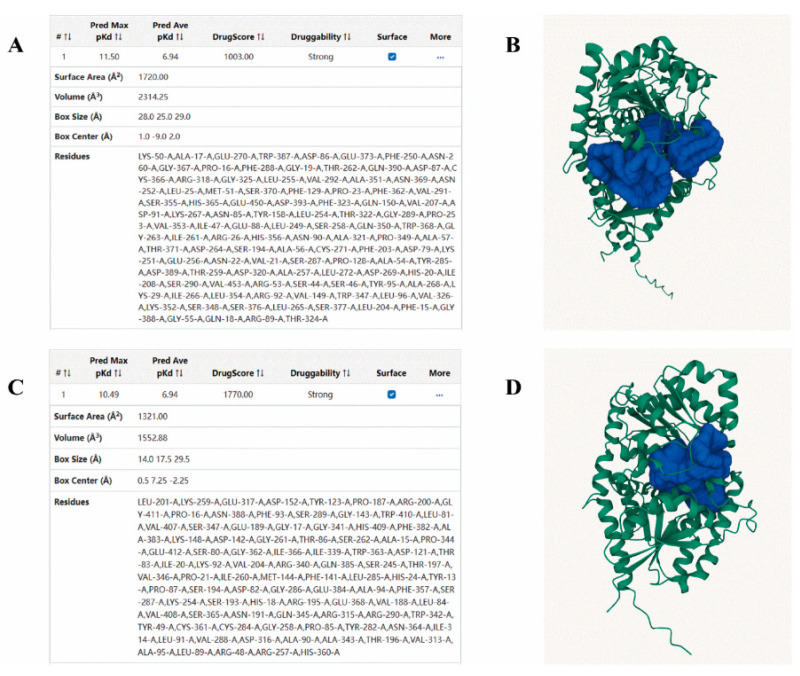
Three-dimensional protein structures and protein binding pocket prediction of *Ct*UGT4 and *Ct*UGT76; (**A**) protein binding pocket prediction of *Ct*UGT4; (**B**) three-dimensional protein structure prediction of *Ct*UGT4; (**C**) protein binding pocket prediction of *Ct*UGT76; (**D**) three-dimensional protein structure prediction of *Ct*UGT76.

All transcripts obtained from transcriptome sequencing at eight timepoints, within one day of full flowering, were compared using six major databases (NR, Swiss-Prot, Pfam, COG, GO, and KEGG), and were annotated as UDP-glycosyltransferase in each database. There were 172 genes in total, and the specific gene names and annotations are shown in Supplementary Table S1. Among the differential genes, the *UGT* genes were then analyzed, as shown in Figure 1. There were 5 *UGT* genes exhibiting significant expression differences between 9:00 and 15:00. Two of these genes showed significantly lower expression at 15:00 compared to 9:00, which was the same pattern noted for most of the flavonoids, including *CtUGT4* and *CtUGT76*. Alphafold2 was used to predict the three-dimensional protein structures of *Ct*UGT76 and *Ct*UGT4, and then CavityPlus was used to predict their protein binding pockets. Interestingly, we found that *Ct*UGT4 has a binding pocket nearly 1.5 times the size of *Ct*UGT76; thus, *Ct*UGT4 was chosen for further functional studies (Figure 2).

### 2.2. Expression and Purification of CtUGTs-HIS and CtUGTs-GST Proteins

Using the safflower cDNA library as a template, the *CtUGT4* gene PCR product was cloned and connected to the linearized vectors pET32a and pGEX-6P-1. The recombinant plasmid was transformed into *Escherichia coli* T1, and the transformants were selected on a plate containing 100 mg/L of ampicillin. The results of bacterial liquid PCR are shown in the Figure 1.

**Figure 3:**
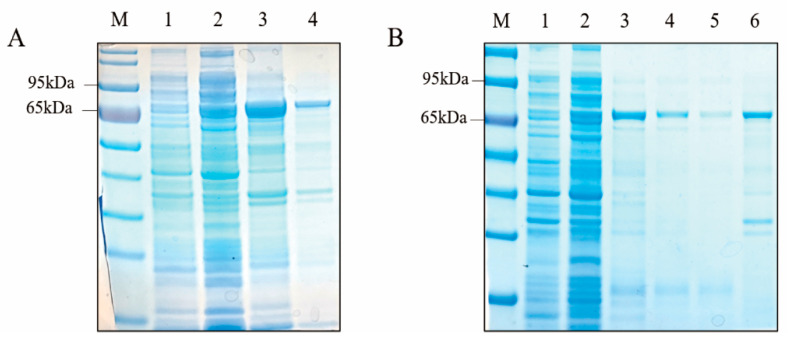
SDS-PAGE electrophoresis diagram of *Ct*UGT4-HIS protein-induced expression and purification; (**A**) electrophoresis diagram of *Ct*UGT4-HIS protein-induced expression; 1: uninduced protein; 2: soluble protein; 3, 4: insoluble protein. (**B**) Electrophoresis diagram of *Ct*UGT4-HIS protein purification. 1: Uninduced protein; 2: soluble protein; 3–6: purified protein.

IPTG was used to induce the expression of *Ct*UGTs-HIS protein. The *Ct*UGT4 protein was approximately 55.87 kDa, the fusion HIS protein was about 18 kDa, and the molecular weight of the induced protein was consistent (Figure 3A). Using BeyoGold™ HIS-tag Purification Resin for protein purification, the purified *Ct*UGT4-HIS protein was obtained (Figure 3B).

**Figure 4:**
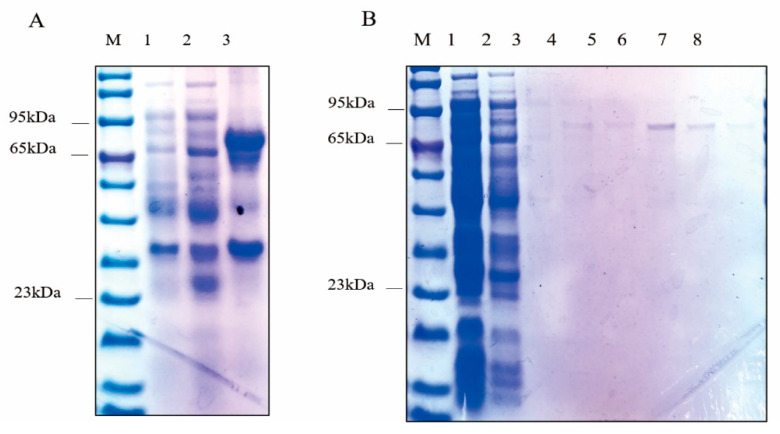
SDS-PAGE electrophoresis diagram of *Ct*UGT4-GST protein-induced expression; (**A**) electrophoresis diagram of *Ct*UGT4-GST protein induced expression; 1: uninduced protein; 2: soluble protein. 3: insoluble protein. (**B**) Electrophoresis diagram of *Ct*UGT4-GST protein purification; 1: uninduced protein; 2: soluble protein; 3–8: purified protein.

IPTG was used to induce the expression of *Ct*UGTs-GST protein. The *Ct*UGT4 protein was approximately 55.87 kDa, the fusion GST protein was about 26 kDa, and the molecular weight of the induced protein was consistent (Figure 4A). Using BeyoGold™ GST-tag Purification Resin for protein purification, the purified *Ct*UGT4-GST protein was obtained (Figure 4B).

### 2.3. Enzyme Activity Reaction and Detection

**Figure 5:**
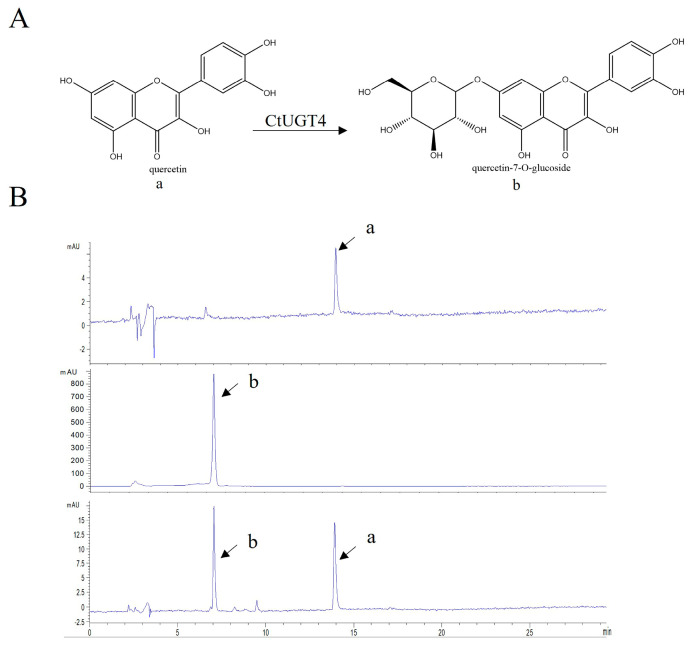
*Ct*UGT4 catalyzes quercetin to form quercetin-7-*O*-glucoside. (**A**) The substrate and product compounds are shown: a−quercetin; b−quercetin-7-*O*-glucoside. (**B**) The HPLC pattern displays the reaction progress, with the enzyme and without the enzyme. From top to bottom: substrate compound in reaction buffer, product compound in reaction buffer, catalytic reaction with the enzyme.

**Figure 6:**
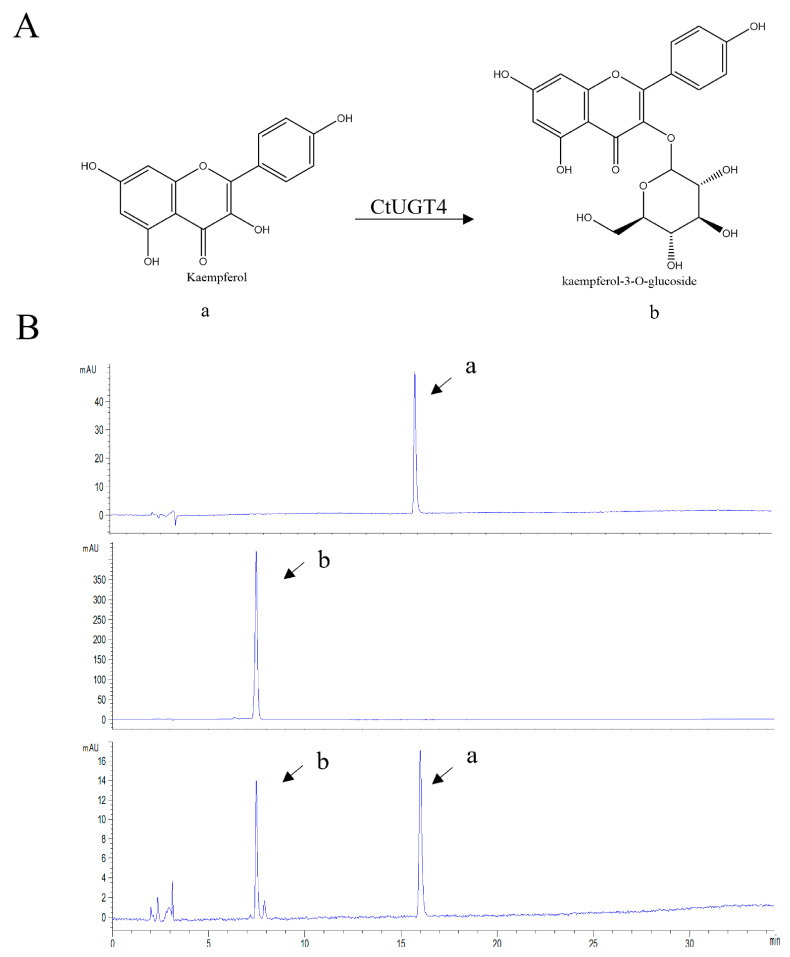
*Ct*UGT4 catalyzes kaempferol to form kaempferol-3-*O*-glucoside. (**A**) The substrate and product compounds are drawn: a−kaempferol; b−kaempferol-3-*O*-glucoside. (**B**) The HPLC pattern shows the reaction progress, with the enzyme and without the enzyme. From top to bottom: substrate compound in reaction buffer, product compound in reaction buffer, catalytic reaction with the enzyme.

To verify the conjecture that *Ct*UGT4 possesses the function of flavonol-*O*-glycosyltransferase, the enzyme activity of the *Ct*UGT4 protein was detected in vitro. Using quercetin and kaempferol as substrates and UDP-glucose as the sugar donor, the results showed that *Ct*UGT4 can catalyze quercetin to form quercetin-7-*O*-glucoside (Figure 5), and also catalyze kaempferol to kaempferol-3-*O*-glucoside (Figure 6). In order to further explore the selectivity of *Ct*UGT4 protein to catalytic substrates, a variety of flavonoids were selected as substrates, including the flavones—apigenin, baicalein, 7-Hydroxyflavone, 6-Hydroxyflavone; the dihydroflavone—naringenin; the chalcone—naringenin chalcone; and the chalcone glycoside—phlorizin. The results showed that *Ct*UGT4 exhibited substrate heterogeneity and glycosyltransferase activity for a variety of flavonoids (Supplementary Figure S2). The results also showed that the catalytic effect of the protein expressed in tobacco on kaempferol was at position 3.

An interesting observation from the results of the in vitro experiments was that the heterologously expressed proteins of *Ct*UGT4 in *E. coli* showed different catalytic sites for kaempferol and quercetin, indicating that this might be due to a lower regioselectivity of the heterologously expressed proteins for the glycosylation sites.

### 2.4. Enzyme Activity Detection of Transiently Expressed Proteins in Tobacco

In order to explore whether the regioselectivity of proteins expressed in *E. coli* also exists in plants, we conducted both in vivo and in vitro enzyme activity detection in tobacco.

Using the safflower cDNA library as a template, the *CtUGT4* gene PCR product was cloned and connected to the linearized vectors pEAQ-HT. The recombinant plasmid was transformed into *E. coli* T1, and the transformants were selected on a plate containing 100 mg·L^−1^ of kanamycin. The results of bacterial liquid PCR are shown in the figure (Supplementary Figure S3).

**Figure 7:**
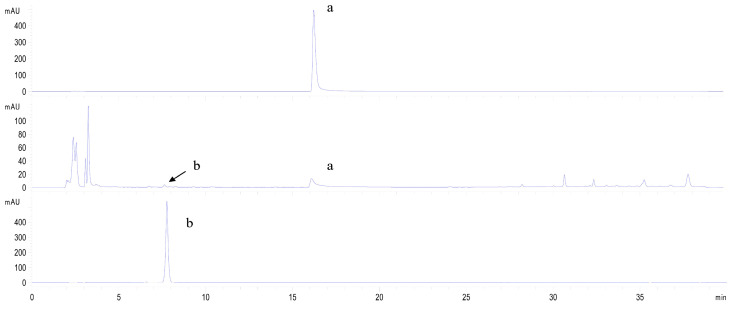
*Ct*UGT4 catalyzes kaempferol to form kaempferol-3-*O*-glucoside in tobacco leaves. The HPLC pattern displays the reaction progress, with the enzyme and without the enzyme. From top to bottom: substrate compound in reaction buffer; catalytic reaction with the enzyme; product compound in the reaction buffer. a: kaempferol; b: kaempferol-3-*O*-glucoside.

As shown in Figure 7, the *Ct*UGT4 protein expressed in tobacco leaves injected with quercetin and kaempferol as substrates, respectively, could catalyze kaempferol to kaempferol-3-*O*-glucoside.

**Figure 8:**
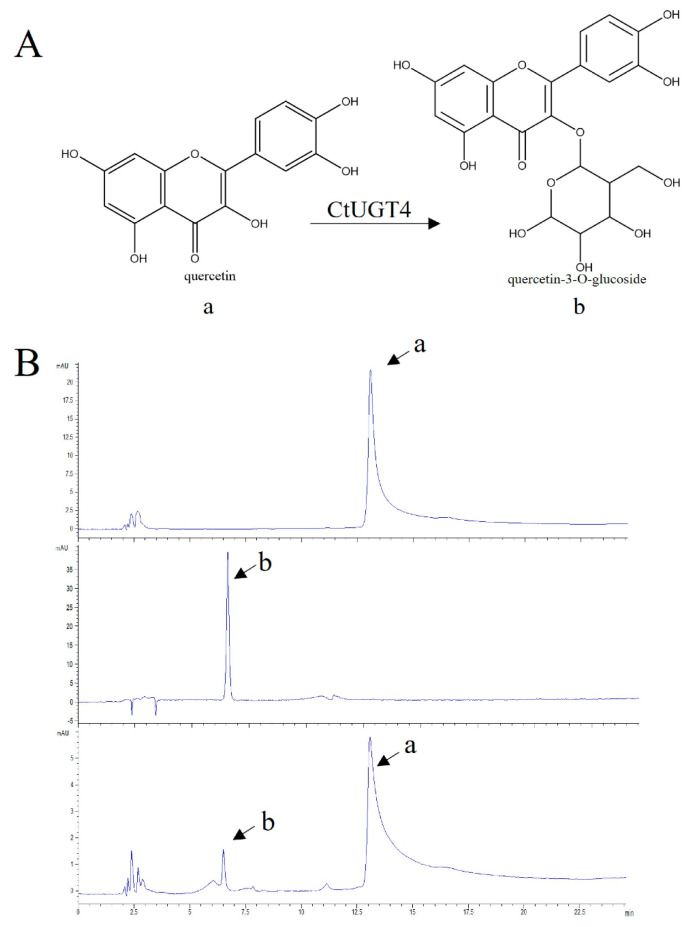
Tobacco protein transiently expressing *Ct*UGT4 catalyzes quercetin to form quercetin-3-*O*-glucoside in vitro. (**A**) Substrate and product compounds are shown: a−quercetin; b−quercetin-3-*O*-glucoside. (**B**) The HPLC pattern displays the reaction progress, with the enzyme and the without enzyme. From top to bottom: substrate compound in reaction buffer, product compound in reaction buffer, catalytic reaction with the enzyme.

**Figure 9:**
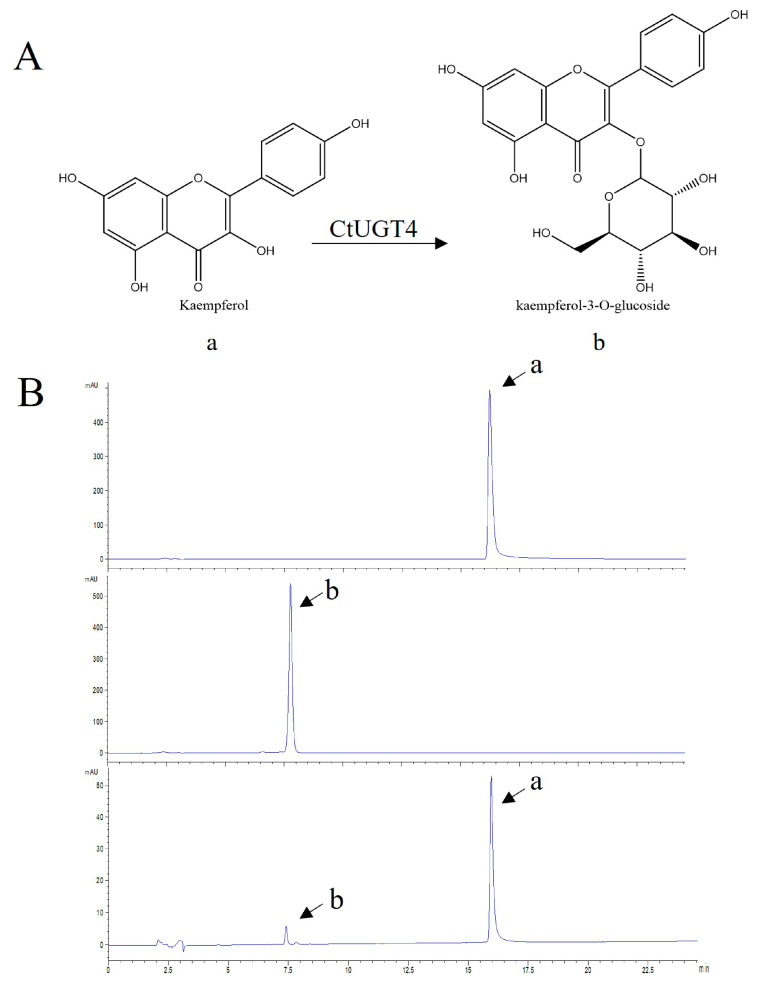
Tobacco protein transiently expressing *Ct*UGT4 catalyzes kaempferol to form kaempferol-3-*O*-glucoside in vitro. (**A**) The substrate and product compounds are shown: a−kaempferol; b−kaempferol-3-*O*-glucoside. (**B**) The HPLC pattern displays the reaction progress, with the enzyme and without the enzyme. From top to bottom: substrate compound in reaction buffer, product compound in reaction buffer, catalytic reaction with the enzyme.

Tobacco protein was extracted after transiently expressing *Ct*UGT4 from tobacco, and the enzyme activity was detected in vitro. As shown in Figure 8 and Figure 9, *Ct*UGT4 could catalyze quercetin to quercetin-3-*O*-glucoside, and catalyze kaempferol to kaempferol-3-*O*-glucoside. The *Ct*UGT4 protein expressed in tobacco in vitro had no regioselectivity for either quercetin or kaempferol at the 3-position catalytic site, which differed from the results obtained for the protein expressed in *E. coli* and the results for the in vivo enzymatic activity in tobacco. It is speculated that this may be due to the effect of different environments on the regioselective priority of the *Ct*UGT4 protein when it expressed.

### 2.5. Overexpression Plant Breeding

Due to the complexity of the in vitro enzymatic reaction results, it was neccessary to validate the function of *Ct*UGT4 protein in safflower in vivo.

To explore whether *Ct*UGT4 was involved in the synthesis of flavonoid glycosides in safflower, transgenic safflowers overexpressing *CtUGT4* under the CaMV 35S promoter were obtained using the pollen tube passage method. Through PCR detection of genomic DNA, the 14 *CtUGT4* overexpression-positive transgenic plants were screened out, and there was almost no difference in plant morphology and growth status between the WT (wild-type safflower line) and the transgenic safflowers.

**Figure 10:**
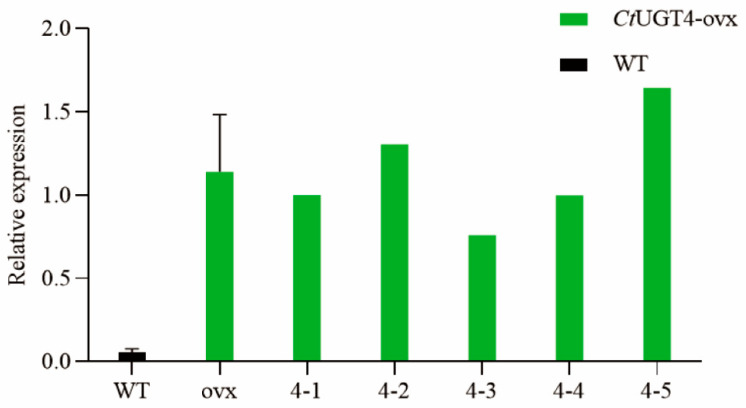
Transcription level of *Ct*UGT4 in *Ct*UGT4-overexpressed plants. Error bars indicate SE (*n* = 5).

Relative fluorescence quantification was used to detect overexpression in plants. The expression level of 4–5 was the highest in the *UGT4*-ovx plants, which was 28.96 times higher than that of wild type. The 4-2 plants were 22.79 times higher; the 4-1 and 4-4 plants were 17 times higher; and the 4-3 plants were 12 times higher than the levels found in the wild-type plants; thus, the *CtUGT4* plants with an overexpression above five were selected for further detection of metabolic content (Figure 10).

### 2.6. Detection of Metabolic Content in Overexpressed Plants

**Figure 11:**
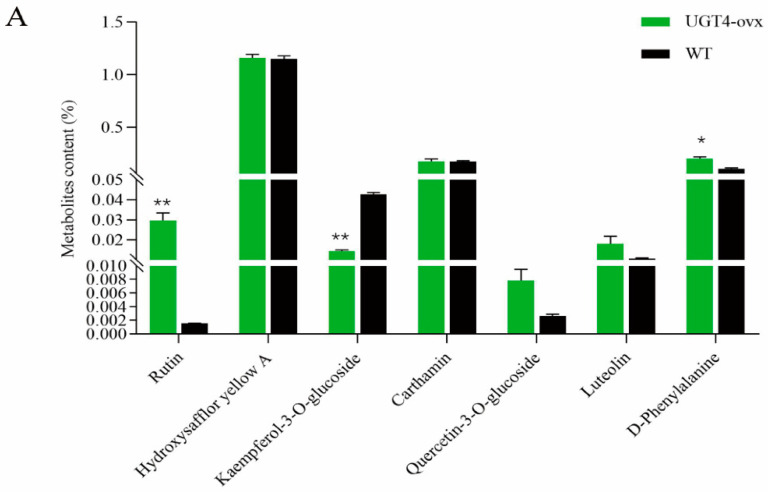

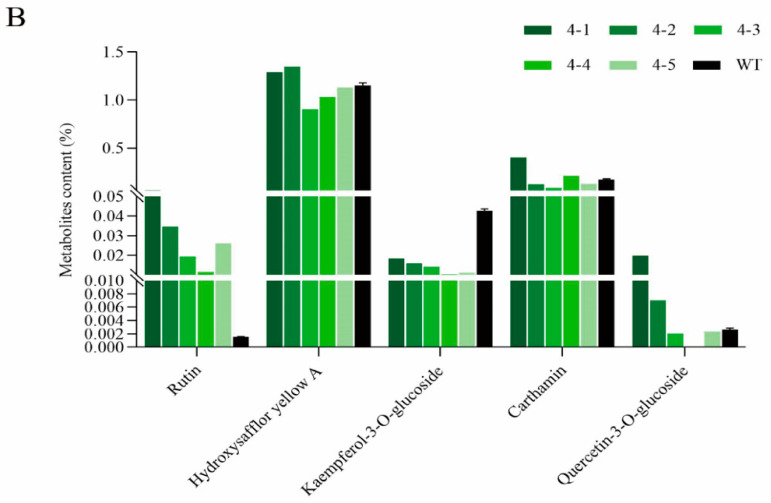
Accumulation of flavonoid glycosides in *Ct*UGT4-transgenic plants. Error bars indicate SE (*n* = 5). * *p* ≤ 0.05; ** *p* ≤ 0.01. (**A**) Average metabolites contents of *Ct*UGT4-overexpressed plants compared with wild type plant. (**B**) Metabolites contents of five *Ct*UGT4-overexpressed plants and one wild type plant individually.

The test results for the flavonoid content in the *Ct*UGT4-overexpressed plants showed that the content of quercetin-3-*O*-rutinoside (rutin) was significantly increased after *Ct*UGT4 overexpression, and the content of quercetin-3-*O*-glucoside also tended to increase. The content of kaempferol-3-*O*-glucoside was significantly reduced. However, the accumulation of quinone chalcone glycoside Hydroxysafflower Yellow A (HSYA) and carthamin did not change. As shown in Figure 11, compared with the safflower control group, after *Ct*UGT4 overexpression, the content of rutin increased by 6.3–39.6 times, while the content of kaempferol-3-*O*-glucoside decreased by 57–76%.

The above conclusions suggested that *Ct*UGT4 might be a flavonol-*O*-glycosyltransferase, which has a higher affinity with the quercetin substrate, directing the metabolic flow to the quercetin-*O*-glycoside branch, and *Ct*UGT4 exhibited substrate heterogeneity for sugar donors in safflower.

The detection results for safflower were different from the results of *Ct*UGT4 expression in tobacco. These results might indicate that *Ct*UGT4 possesses two catalytic sites for the same substrate, but is subject to different in vivo environments, as well as changes in exogenous induction conditions, producing different results.

## 3. Discussion

UGT affects the accumulation level of plant secondary metabolites through the glycosylation during plant secondary metabolism. However, in many medicinal plants, the roles of flavonoid glycosyltransferases are not clear. Safflower is a well-known medicinal plant in China. This research validates the function of the *Ct*UGT4 in the biosynthesis of flavonoids, exerting important physiological effects in safflower.

*Ct*UGT4 protein can catalyze quercetin to quercetin-7-*O*-glucoside, and kaempferol to kaempferol-3-*O*-glucoside, and a series of flavones, flavonols, dihydroflavones, chalcones, and chalcone glycosides can all serve as catalytic substrates for *Ct*UGT4 in vitro, indicating the substrate heterogeneity of *Ct*UGT4. Our results were consistent with the characterization of substrate heterogeneity of UGT genes previously reported in other species [36,37,38]. The substrate heterogeneity of UGT proteins may be due to the topology of the binding pocket in the secondary protein structure. The partially disordered loop that constitutes the binding pocket endows it with higher flexibility and larger acceptor binding pockets [39]. This also reminds us that enzyme-based biosynthesis exhibits higher stereoselectivity and regioselectivity than does chemical synthesis [40], as the volume of the binding pocket can be altered by inducing mutations in protein residues, thereby yielding the specifically target metabolites [41]. Meanwhile, the *Ct*UGT4 protein heterologously expressed in *E. coli* catalyzed the production of quercetin-7-*O*-glucoside from quercetin, while the *Ct*UGT4 protein heterologously expressed in tobacco catalyzed the production of quercetin-3-*O*-glucoside from quercetin. The glycosylation sites catalyzed by *Ct*UGT4 are different under different expression systems, and this phenomenon also exists in other plants. Among the 91 recombinant UGTs of *Arabidopsis thaliana* tested for quercetin activity, 15 UGTs showed the ability to glycosylate two or more sites [23]. Tea (*Camellia sinensis*) *Cs*UGT73A20 possesses catalytic sites for both position-3 and position-7 quercetin and can catalyze two products at the same time. The priority of the catalytic sites is different under different pH conditions [42]. So far, the explanation of the regioselectivity of UGTs in regards to the catalytic site has focused mainly on their key amino acid residues. The studies of protein chimeras with different preferences for catalytic sites and the results of site-directed mutagenesis have also shown that the affinity of UGT for these sites can be directly changed [41,43].

As a molecular modification reaction widely present in plants, glycosylation can affect both the amount and the pharmacological activity of endogenous products by changing the solubility and other properties of the end products [44]. The overexpression of glycosyltransferases in plants can increase the content of their secondary metabolites. Yin et al. overexpressed six *Gm*UGTs in soybean and Arabidopsis and found that the flavonoid content in both plants can be significantly increased [45]. Tang et al. overexpressed 3GT in the callus of *Saussurea involurcrata* and detected that the flavonoid content increased by 2.06 times [46]. The content of quercetin-3-*O*-rutinoside (rutin) was significantly increased in *CtUGT4*-overexpressed safflower, and the content of quercetin-3-*O*-glucoside also had a tendency to increase, while kaempferol-3-*O*-glucoside was reduced. Our results indicated that *Ct*UGT4 may be a flavonol-*O*-glycosyltransferase, and it has a higher affinity for quercetin substrate than kaempferol substrate, directing the metabolic flow to the quercetin-*O*-Glycoside branch. The increase in *Ct*UGT expression can promote the improvement of a class of flavonol glycosides in safflower, which makes it possible to directionally improve the quality of safflower, providing a new idea for cultivating safflower varieties with a higher content of effective metabolites and therefore, a higher economic value.

Meanwhile, in the transient expression system of tobacco, *Ct*UGT4 can catalyze kaempferol to kaempferol-3-*O*-glucoside, which is different from the decrease in kaempferol-3-glucoside noted in safflower. These differences may lie in the complexity of the in vivo environment, as well as in the differences in the subcellular localization of the substrates and compounds, etc., but the exploration of the specific reasons and mechanism still requires further research.

In summary, this study provides a preliminary exploration of the *O*-GT of the biosynthetic pathway of flavonoids in safflower. In addition, the *C*-GT gene, which plays a direct role in regulating the biosynthetic pathway of another active component of safflower, quinone chalcone, requires further exploration.

## 4. Materials and Methods

### 4.1. Plant Materials and Chemicals

*C. tinctorius* L. is a strain from Weishan, Yunnan (ZHH0119), and its seeds were collected from the National Germplasm Resource Bank in the Academy of Agricultural Sciences of Xinjiang. Professor Guo Meili identified it as safflower (*Carthamus tinctorius* L.). It has been repeatedly purified in our laboratory. The plant was grown under circadian lighting (16 h/8 h light–dark cycle) in a 25 °C ± 4 °C greenhouse at the Naval Medical University (Shanghai, China). The voucher specimen is SMMU171201, held by the Naval Medical University.

Carthamin was extracted by our laboratory, and the chemical standards were purchased from Shanghai Source Leaf Biological Technology Company (Shanghai, China) and Sigma-Aldrich (Shanghai, China). All organic solvents used for high-performance liquid chromatography (HPLC) were obtained from Sinopharm Chemical Reagent Co., Ltd. (Shanghai, China).

### 4.2. In Vitro Expression Vector Construction

For the construction of the vectors for *E. coli* prokaryotic expression system, the target gene fragment was cloned with the homology arms of the pET32a vector. The PCR reaction program is as follows: the gene fragments are subjected to temperatures of 95 °C for 3 min, followed by 35 cycles of 95 °C for 15 s, 55 °C for 15 s, 72 °C for 1 min, and finally 72 °C for 5 min. The pET32a plasmid was digested with restriction endonucleases NcoI and SacI, the PCR product and the digested product were subjected to agarose gel electrophoresis, the gel was recovered, and the fragments were connected to the linearized vector using the seamless cloning method. *E. coli* T1 was transformed, and the culture was expanded, the plasmid was extracted, and sequencing was performed. The correctly sequenced plasmid was transformed into expression-competent Rosetta (DE3), and single-clonal colonies were selected. Transformed *E. coli* T1 and Rosetta (DE3) competent cells were purchased from Shanghai Weidi Biotechnology Co., Ltd. (Shanghai, China) After the liquid expansion culturing, the positive clones were identified using bacterial liquid PCR, and the primers used were T7 and T7t primers.

For the construction of the tobacco transient expression vector, the primers were AgeI-*CtUGT4*: ctgcccaaattcgcgaccggtATGGCTTCCAAACAAGGCCT, *CtUGT4*-XhoI: accagagttaaaggcctcgagATTGGGCACCGGACATCG. The construction method was the same as that used for the above-mentioned *E. coli* expression system vector.

### 4.3. Protein Expression and Purification

Protein expression was induced with IPTG. Used 500 mL of LB medium, containing kanamycin (100 mg·L^−1^), to amplify the above-mentioned positive clones, they were subjected to 37 °C, 200 rpm to OD_600_ of 0.4–0.5. After cooling, IPTG was added to yield a final concentration of 0.1 mM, and the solution was subjected to 16 °C at 60 rpm for 12 h to induce protein expression. The induced bacterial liquid was centrifuged at 4000 rpm for 5 min to collect the bacterial cells. The liquid was then washed once with PBS buffer, the cells were again collected by centrifugation, and the cells were then resuspended in lysis buffer with 1/10 of the volume of the original cell solution, and protease inhibitors were added. An ultrasonic breaker with a working power of 30% was employed for this process, working for 1.9 s and resting for 3.1 s, for 20–60 min until the bacterial liquid became clear. The liquid was then subjected to 4 °C at 13,000 rpm, and centrifuged for 20 min to separate the supernatant and precipitate.

For protein purification, the protein supernatant was filtered with a 0.45 µm microporous membrane, 10 mL of BeyoGold^TM^ His-tag Purification Resin (Beyotime, Shanghai, China) was obtained with a pipette fitted with a minus tip, and the sample was equilibrated 5 times using a lysis buffer. The filtered protein supernatant was mixed with His-tag Purification Resin and combined in a shaker at 4 °C for 5 h. The mixed solution was then poured into the purification column, the cap was opened to let it flow out, and the effluent was collected. The effluent was again poured into the purification column, allowed to flow out, washed 5 times with 1 column volume of washing solution until no protein flowed out, using 1/2 column volume of eluent to elute the solution 10 times, and the eluate was collected.

For protein concentration, the protein ultrafiltration tube was soaked in ultrapure water, placed on ice for 20 min, and the pure water was poured out and added to the protein solution. The mixture was centrifuged at 5000 rpm at 4 °C for 10–20 min, and concentrated to 500 µL. A total of 1 mL of 50 mM Tris-HCl (pH 7.4) was added, the mixture was centrifuged at 5000 rpm at 4 °C for 10 min, and the process was repeated three times. The concentrated protein was aspirated with a pipette tip and stored it at −70 °C for later use.

### 4.4. Enzyme Activity Reaction and Detection

The standard compound was accurately weighed and dissolved in DMSO to reach 100 mM and then stored at −20 °C for later use. UDP-glucose was accurately weighed and dissolved in 50 mM Tris-HCl (pH 7.4) to reach 100 mM, and it was prepared and used immediately. The reaction system was prepared (20 µL UGT protein, 0.25 µL substrate, 0.5 µL UDP-glucose, 50 mM Tris-HCl (pH 7.4) 80 µL), reacted at 30 °C for 12 h, and 200 µL of ice methanol was added to terminate the enzymatic reaction. The above enzyme activity reaction product was centrifuged at 12,000 rpm for 5 min, and the supernatant was absorbed for HPLC sample injection. The HPLC reaction conditions were as follows: injection volume: 20 µL; chromatographic column: Agilent 1100 series C18 column (5 µm, 4.6 × 250 mm); mobile phase A: 0.1% formic acid; mobile phase B: acetonitrile with 0.1% formic acid; separation gradient: 0–2 min, 5%B; 2–2.5 min, 5–15%B; 2.5–7.5 min, 15%B; 7.5–8 min, 15–20%B; 8–10 min, 20–21%B; 10–18 min, 21–95%B; 18–20 min, 95%B; flow rate: 1 mL·min^−1^; and column temperature: 25 °C.

### 4.5. Transient Expression in Tobacco

The plasmids confirmed by sequencing were transformed into GV3101 agrobacterium competent cells, purchased from Shanghai Weidi Biotechnology Co., Ltd., and spread on LB solid medium containing kanamycin (100 mg·mL^−1^) and rifampin (25 µg·mL^−1^). The liquid medium was shaken at 28 °C for 12 h at 200 rpm, and the positive colonies were identified. The positive bacterial solution identified to be above to 5 mL of LB liquid medium containing kanamycin (50 mg·mL^−1^) and rifampin (25 µg·mL^−1^) was added, shaken overnight at 28 °C at 200 rpm, centrifuged at 5000× *g* for 5 min, removed from the medium, and resuspended in buffer solution (10 mM MES, 10 mM MgCl_2_, 150 µM acetosyringone, pH 5.6) to reach an OD_600_ of 0.6.

A 1 mL syringe was used to obtain the above bacterial solution, and it was injected from the back unto the tobacco, and culturing was continued for 4 or 5 days. The injected leaves were collected, quick-frozen in liquid nitrogen, and stored in a −80 °C refrigerator.

### 4.6. Detection of Enzyme and Metabolite Activity of Tobacco

For the detection of enzyme activity in tobacco, 4 days after the injection of protein expression bacterial solution, the tobacco leaves were injected with 250 µM of quercetin, kaempferol, or apigenin in the same area, and after continued culturing for 1 day, the collected leaves were quick-frozen with liquid nitrogen and stored in a −80 °C refrigerator.

For tobacco enzyme activity detection in vitro, polyvinylpyrrolidone and the tobacco leaves collected 4 days after the injection of the protein expression bacterial solution were mixed, ground for 2 min with a tissue grinder at 25 Hz, resuspended in 1 mL of 100 mM Tris-HCl solution (10% glycerol, 1 mM PMSF, 10 mM β-mercaptoethanol), and mixed until homogeneity was reached. The mixture was then placed it on ice for 1 h, centrifuged at 12,000× *g* for 10 min at 4 °C, and the supernatant was absorbed. The reaction system (100 µL) contained 0.5 mM of substrate (quercetin, kaempferol or apigenin) and 1 mM of UDP-glucose and protein supernatant, which was incubated at 30 °C for 3 h. The reaction was terminated by adding 100 µL of acetonitrile and freeze-drying. The lyophilized enzyme reaction product was resuspended in 200 µL acetonitrile, and the metabolites were extracted with 60% methanol. For the detection by HPLC, the method was the same as that used for the detection of enzyme activity.

### 4.7. Vector Construction and Cultivation of Safflower Transgenic Plants

The vector of the *E. coli* prokaryotic expression system and cloned the target gene fragment was constructed with a homology arm. The PCR reaction program consisted of subjecting the mixture to 95 °C for 3 min, followed by 35 cycles of 95 °C for 15 s, 55 °C for 15 s, 72 °C for 1 min, and finally, 72 °C for 5 min.

The PMT39 plasmid (pCAMBIA-1380-CaMV35S-MCS-EGFP-NOS) was digested with NcoI restriction endonuclease. The PCR product and the digested product were isolated by agarose gel electrophoresis, the electrophoresis gel was recovered, and the seamless cloning method was used to ligate the fragments with the linearized vector. The ligated product was transformed into *Escherichia coli* T1, and the plasmid was extracted after expanding the culture.

The constructed vector was sent to Sangon (Shanghai, China) for sequencing, and Megalign software (Version 7.1.0) was used to compare the sequencing results. After confirming that it was the target gene, the vector was transformed into GV3101 agrobacterium competent cells, a monoclonal colony was selected, and the positive clone was identified by PCR of the liquid expanded culture. The primers were: IDF: ATCTCTCTCGAGCTTTCGCGG, IDR: TCAGGGTCAGCTTGCCGTAG. The agrobacterium was transferred into safflower to produce safflower overexpressing *CtUGT4* using the pollen pipeline method. After the transformed safflower was fully mature, the seeds were harvested and planted. After flowering, the safflower florets, with the ovary removed, were collected, and their genomic DNA was extracted. Primers were designed for the identification of transgenic positive plants; the upstream identification primer was on the 35S promoter, and the downstream identification primer was on the ORF (35SIDF: CGACAGTGGTCCCAAAGAT, OvxU4IDR: AAGGTGACAAGAAGGTTA).

### 4.8. Detection of Metabolite Content in Transgenic Safflower

Using our previous method, sample pretreatment was carried out on the overexpressed safflower identified as positive [47]. UPLC-Q-TOF/MS was used to detect the content of the following compounds: D-phenylalanine, kaempferol, quercetin, naringenin, apigenin, luteolin, rutin, kaempferol-3-*O*-β-d-rutinoside, kaempferol-3-*O*-β-d-glucoside, quercetin-3-*O*-β-d-glucoside, scutellarein, hydroxysafflower yellow A (HSYA), andcarthamin. The standard curve method was used to detect the compound content in the sample, and the standard curve is shown in Supplementary Table S2. The chromatographic and mass spectrometric methods used previously were employed in this study [48]. The Agilent 6538 Accurate Mass Quadrupole Time-of-Flight MS and Agilent 1290 Infinity LC System (Agilent, Santa Clara, CA, USA) devices were used for ultra-high performance liquid chromatography using quadrupole time-of-flight mass spectrometry (UHPLC-QTOF-MS) analysis. An XBridgeTM BEH C18 column (2.5 µm, 2.1 mm × 100 mm; Waters, Milford, MA, USA) was used for chromatographic separations.

## 5. Conclusions

In this study, an *O*-glycosyltransferase, *Ct*UGT4, involved in the biosynthesis of flavonoids in safflower, was characterized, and its function was verified, both in vitro and in vivo. The results proved that *Ct*UGT4 exhibited substrate promiscuity and stereospecificity, as well as the ability to catalyze the *O*-glycosylation of various flavonoids. It is worth noting that *Ct*UGT4 obtained by different expression hosts selected different glycosylation sites for the same substrate. Furthermore, the overexpression of *Ct*UGT4 in safflower confirmed that its high expression level resulted in increasing the content of a specific class of flavonol glycosides in safflower. The results partly explained the glycosyl modification function of flavonoids in safflower, and also provide a valuable reference for the related study of homologous genes in other plants.

## Data Availability

Data is contained within the article or Supplementary Materials.

## Supplementary Materials

The following supporting information can be downloaded at: https://www.mdpi.com/article/10.3390/molecules28227613/s1. Figure S1: Electropherogram of CtUGT4 fragment cloning, CtUGT4-pET32a and CtUGT4-pGEX-6P bacterial solution PCR. Figure S2: CtUGT4 is catalytically active towards a variety of flavonoids. HPLC pattern display reaction progresses with enzyme and without enzyme. a: substrate b: products of enzymatic reactions. (A) from top to bottom: apigenin as substrate in reaction buffe, catalytic reaction with enzyme. (B) from top to bottom: baicalein as substrate in reaction buffe, catalytic reaction with enzyme. (C) from top to bottom: 7-Hydroxyflavone as substrate in reaction buffe, catalytic reaction with enzyme. (D) from top to bottom: 6-Hydroxyflavone as substrate in reaction buffe, catalytic reaction with enzyme. (E) from top to bottom: 7,4-Dihydroxyflavone as substrate in reaction buffe, catalytic reaction with enzyme. (F) from top to bottom: naringenin as substrate in reaction buffe, catalytic reaction with enzyme. (G) from top to bottom: naringenin chalcone as substrate in reaction buffe, catalytic reaction with enzyme. (H) from top to bottom: phlorizin as substrate in reaction buffe, catalytic reaction with enzyme. Figure S3: Bacterial liquid PCR of CtUGT4-pEAQ-HT. Table S1: The specific gene names and annotations of 172 genes annotated as UDP-glycosyltransferase. Table S2: Linear regression data of the investigated flavonoids.
