# Supplementary material for: A Muti-Substrate Flavonol O-glucosyltransferases from Safflower
Source: Molecules. 2023 Nov 15;28(22):7613. doi: 10.3390/molecules28227613 (PMC10674463; doi:10.3390/molecules28227613)
Supplement: Supplementary file 1 [file molecules-28-07613-s001.zip › molecules-2682217 - Supplementary Figures.pdf]

molecules

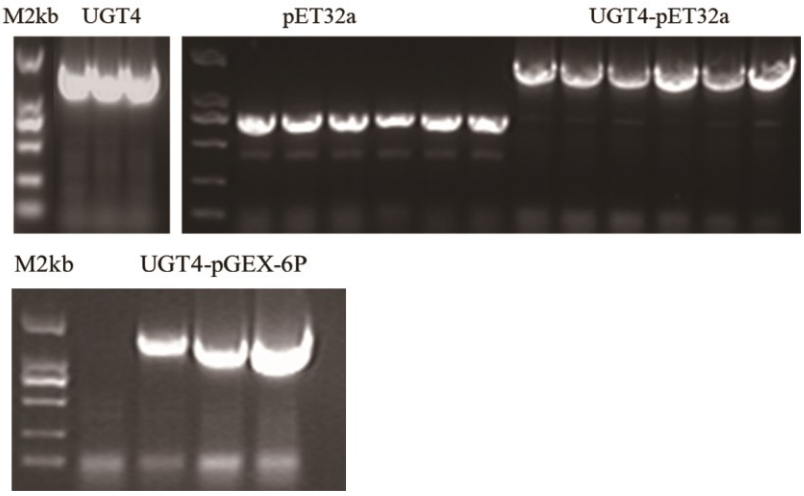

Figure S1. Electropherogram of CtUGT4 fragment cloning, CtUGT4-pET32a and CtUGT4-pGEX-6P bacterial solution PCR.

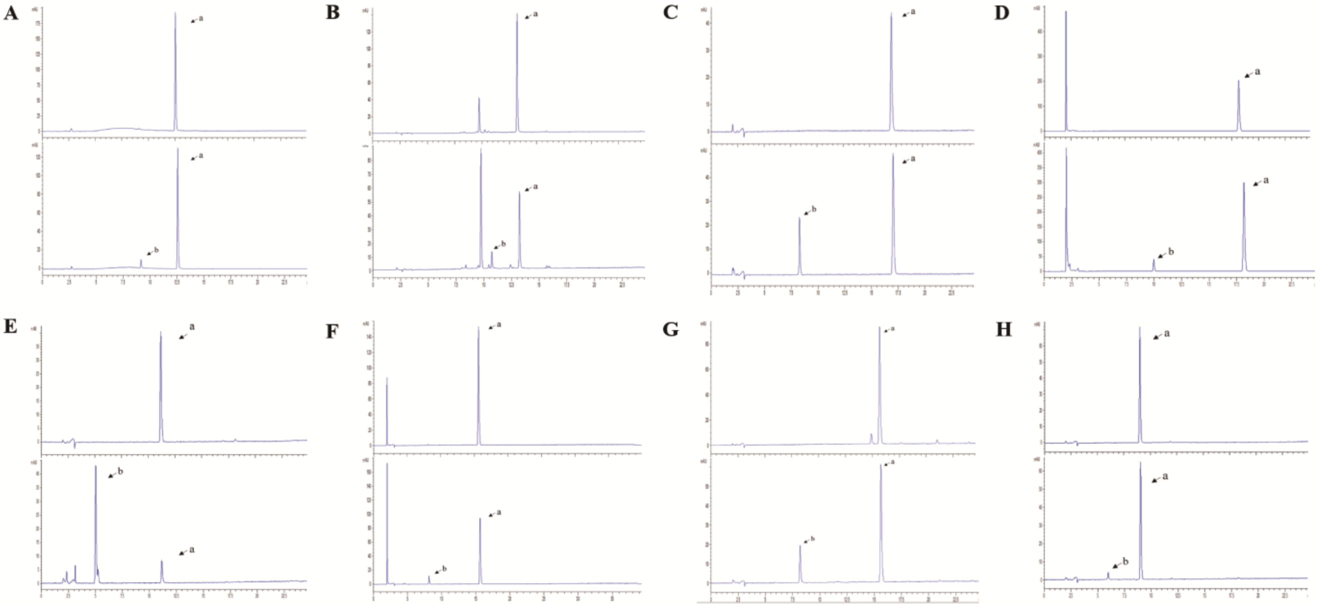

Figure S2. CtUGT4 is catalytically active towards a variety of flavonoids. HPLC pattern display reaction progresses with enzyme and without enzyme. a: substrate b: products of enzymatic reactions. (A) from top to bottom: apigenin as substrate in reaction buffe, catalytic reaction with enzyme. (B) from top to bottom: baicalein as substrate in reaction buffe, catalytic reaction with enzyme. (C) from top to bottom: 7-Hydroxyflavone as substrate in reaction buffe, catalytic reaction with enzyme. (D) from top to bottom: 6-Hydroxyflavone as substrate in reaction buffe, catalytic reaction with enzyme. (E) from top to bottom: 7,4-Dihydroxyflavone as substrate in reaction buffe, catalytic reaction with enzyme. (F) from top to bottom: naringenin as substrate in reaction buffe, catalytic reaction with enzyme. (G) from top to bottom: naringenin chalcone as substrate in reaction buffe, catalytic reaction with enzyme. (H) from top to bottom: phlorizin as substrate in reaction buffe, catalytic reaction with enzyme.

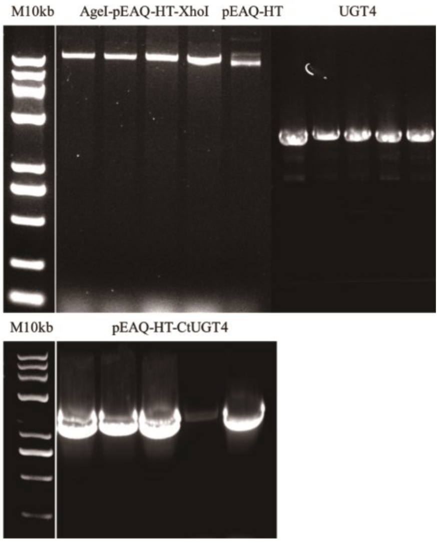

Figure S3. Bacterial liquid PCR of CtUGT4-pEAQ-HT.
